# Clinical Debates in Minimally Invasive Glaucoma Surgeries (MIGSs)

**DOI:** 10.3390/jcm15010240

**Published:** 2025-12-28

**Authors:** Etsuo Chihara

**Affiliations:** 1 Department of Ophthalmology, Shimane University, Enya 693-8501, Shimane, Japan; chiha492001@gmail.com; Tel.: +81-774-45-2060; 2 Sensho-kai Eye Institute, Uji 611-0043, Kyoto, Japan

I am pleased to introduce this Special Issue of the *Journal of Clinical Medicine*, dedicated to “Clinical Debates in minimally Invasive Glaucoma Surgeries (MIGSs)”, which address current and emerging topics in glaucoma surgery.

Glaucoma is one of the leading causes of blindness worldwide [1,2]. Reduction in intraocular pressure (IOP) remains the only evidence-based and modifiable treatment strategy for glaucoma. Medical therapy and laser procedures are considered first-line approaches for lowering IOP; however, when these treatments fail to achieve adequate IOP control, surgical intervention becomes necessary.

Traditionally, trabeculectomy has been regarded as the gold standard surgical treatment for glaucoma. However, this procedure is associated with a high risk of postoperative complications, including bleb leaks, infection, postoperative hypotony, and deterioration of visual acuity [3,4].

To address the shortcomings of trabeculectomy, minimally invasive glaucoma surgeries (MIGSs) have been developed. Over the past decade, substantial advances have been made in understanding the mechanism of IOP reduction, as well as the safety profile and surgical efficacy of MIGS procedures. This Special Issue covers a broad spectrum of topics, including the historical evolution of MIGS, recent trends in surgical decision making, advances in surgical devices and technologies, and tissue responses following MIGS procedures.

There are several types of MIGSs, among which canal-opening surgery (COS) is one of the most widely used subtypes. There has been a long-standing debate regarding the mechanism of action and clinical outcomes of COS. Chihara and Hamanaka (Contribution 1) reviewed the historical development of MIGS and critically discussed the mechanism of action underlying COS. It took considerable time to understand the differences in tissue responses between experimental monkey eyes, where the trabecular opening closes shortly after surgery due to tissue regeneration [5], resulting in limited improvement in outflow facility [6], and human eyes, in which canal opening tends to persist [7] and IOP reduction is maintained over a long period [8]. The long-term debate on the clinical outcome of COS in human eyes is extensively reviewed in a review article of this series (Contribution 1).

There are several types of MIGS. Among them, canal opening surgeries, which originated from trabeculotomy ab externo, have recently evolved and are now widely used for the treatment of mild to moderate open angle glaucoma (OAG). With respect to postoperative visual acuity, there is a clear contrast between COS and trabeculectomy. The advantage of MIGS in preserving and restoring visual acuity was elegantly demonstrated by Kasahara et al. (Contribution 2), which is one of the key reasons COS is increasingly selected for patients with mild to moderate OAG.

An increasing trend in the use of MIGS has been reported in several studies [9,10], and this trend was further confirmed by Iwasaki et al. (Contribution 3) in this Special Issue. The extent of canal opening and the type of device used to open Schlemm’s canal may influence surgical outcomes. Sugihara et al. (Contribution 4) reported that IOP reduction after 120-degree canal opening did not differ significantly from that after 240-degree canal opening using the Tanito microhook. This finding is consistent with the report by Ćwiklińska-Haszcz et al. (Contribution 5), in which IOP reduction after 180-degree canal opening using the GATT procedure was not significantly greater than that achieved with canal opening exceeding 190 degrees. These results also align with findings from other large-scale studies [11]. Nevertheless, caution is warranted, as other investigators have reported superior IOP reduction with 360-degree canal opening compared with sectorial canal opening [12,13]. Given the heterogeneity in sample size, follow-up duration, and surgical techniques across studies, this issue remains an important topic for future investigation. Ćwiklińska-Haszcz et al. (Contribution 5) also reported that the number of previous trabeculectomies and the performance of combined cataract surgery did not significantly affect surgical outcomes, a finding that aligns with some previous reports [14,15]. However, conflicting results have been reported by other investigators with several studies suggesting positive additive effects of combined cataract surgery on IOP reduction [16]. Thus, the impact of combined cataract surgery on the outcome of MIGS remains controversial and warrants further study.

When performing iStent surgery, the type of gonio-lens used can influence the surgical time required for implantation of the iStent inject. W, Fujii et al. (Contribution 6) reported that the use of the Ahmed gonio-lens was associated with more efficient implantation and recommended it for this procedure.

The Hydrus Microstent represents a different class of microstent, designed not only to bypass the trabecular meshwork but also to dilate Schlemm’s canal. Although this device effectively reduces IOP, precise insertion into Schlemm’s canal may be technically challenging. Zimmermann et al. (Contribution 7) investigated postoperative positioning of the Hydrus Microstent using a NIDEK GS-1 automated 360°gonioscope and demonstrated substantial variability in postoperative implant positioning. Even though postoperative IOP was successfully reduced in all cases, the distal tip of the nitinol stent was located behind the trabecular meshwork in five cases, in the anterior chamber in one case, and was not visible in seven cases. The long-term clinical implications of Hydrus malpositioning may be a subject for future studies.

Minimally invasive bleb surgery (MIBS) represents another category of glaucoma surgery that is increasingly applied in patients with moderately severe OAG. With respect to the XEN Gel Stent, the device may be implanted via an ab interno or ab externo approach. Jin et al. (Contribution 8) studied the outcome of XEN in the treatment of refractive glaucoma and reported superior results with the ab externo open-conjunctival approach combined with adjunctive Ologen, compared with simple ab interno implantation.

PreserFlo MicroShunt is another type of MIBS; however, when used in the eyes with exfoliation glaucoma, the probability of surgical success at 48 weeks was approximately 50%, and needed reoperation in 30%. Wakuda et al. (Contribution 9) highlighted this limited success probability in exfoliation glaucoma. The long-term outcomes of MIBS therefore remain an important subject for future studies.

Although MIGS and MIBS are generally considered safe procedures with a lower risk of complications, and their effects on corneal deformation are mild, these are not entirely free of adverse effects. Asaoka et al. (Contribution 10) evaluated corneal stiffness using Corvis ST parameters and demonstrated that corneal stiffness was more significantly reduced in eyes undergoing MIGS compared with those undergoing cataract surgery alone.

Overall, this Special Issue highlights the advantages of MIGS and MIBS procedures; however, surgeons must be fully aware of their respective benefits and limitations. Differences in surgical techniques and clinical outcomes should be carefully considered when applying these procedures in clinical practice.

I extend my sincere gratitude to all authors who contributed to this Special Issue and demonstrated outstanding commitment to advancing this important topic. I hope that this Special Issue serves as a valuable resource and stimulates further research into the surgical treatment of OAG.

## Abbreviations

The following abbreviations are used in this manuscript:

MIGS	Minimally invasive glaucoma surgery
COS	Canal opening surgery
IOP	Intraocular pressure
GATT	Gonioscopy-assisted transluminal trabeculotomy
MIBS	Micro-invasive bleb surgery
OAG	Open-angle glaucoma
